# Physically stable yet biologically sensitive: multiyear ecological dynamics of anoxygenic phototrophs in stably redox-stratified Lake Cadagno

**DOI:** 10.1007/s00027-025-01183-1

**Published:** 2025-04-16

**Authors:** N. Storelli, O. Sepúlveda Steiner, F. Di Nezio, S. Roman, A. Buetti-Dinh, D. Bouffard

**Affiliations:** 1 Department of Environment, Constructions and Design, Institute of Microbiology, University of Applied Sciences and Arts of Southern Switzerland (SUPSI), Via Flora Ruchat-Roncati 15, 6850 Mendrisio, Switzerland; 2 Department of Plant Sciences, University of Geneva, Boulevard d’Yvoy 4, 1205 Geneva, Switzerland; 3 Surface Waters – Research and Management, Eawag, Swiss Federal Institute of Aquatic Science and Technology, Seestrasse 79, 6047 Kastanienbaum, Switzerland; 4 Civil and Environmental Engineering, University of California – Davis, 3155 Ghausi Hall, Davis, CA 95616 USA; 5 National Research Council of Italy – Water Research Institute (CNR-IRSA), Largo Tonolli 50, 28922 Verbania, Italy; 6 Alpine Biology Center Foundation, Via Mirasole 22A, 6500 Bellinzona, Switzerland; 7 Institute of Earth Surface Dynamics, University of Lausanne, Quartier UNIL-Mouline, 1015 Lausanne, Switzerland

**Keywords:** Meromictic Lake Cadagno, Anoxygenic photosynthesis, External environmental factors, Long-term monitoring, Schmidt stability

## Abstract

Lake Cadagno is a meromictic alpine lake characterized by permanent stratification, which creates a permanent anoxic environment that supports the growth of anoxygenic phototrophic sulfur bacteria. The seasonality and interseasonality of these microorganisms were examined over a three-year period (2019–2021) through regular monitoring of the water column. A variety of physical–chemical parameters, including temperature, conductivity, light, oxygen and sulfide concentrations, and the community composition of anoxygenic phototrophic sulfur bacteria in the chemocline were recorded, to observe potential influence of external weather conditions. Our findings indicate that, despite the lake’s consistent physical and chemical stratification, the composition of the anoxygenic phototrophic sulfur bacteria community exhibited notable variations in response to external environmental factors, including changes in rainfall and light irradiance. Specifically, we observed different growth dynamics in the purple (PSB) and green (GSB) sulfur bacteria communities over the three years of monitoring. These variations underscore the complexity of biogeochemical cycles in meromictic lakes and the impact of external environmental factors on this ancestral microbial community dynamics. The results provide valuable insights into the stability of redox-stratified environments, offering a modern analog for ancient aquatic ecosystems. This research emphasizes the importance of long-term regular monitoring to capture interannual dynamics and assess the implications of climate change on such unique ecosystems.

**Supplementary Information:**

The online version contains supplementary material available at 10.1007/s00027-025-01183-1.

## Introduction

The structure of the water column in lakes is primarily influenced by the distribution of temperature and the concentration of dissolved ions, which control the water density and, thereby, stratification (Boehrer and Schultze 2008). The type of stratification is either temporary (holomictic) or permanent (meromictic). Most lakes are holomictic and can be classified as monomictic, dimictic, and polymictic on the basis of the overturning tendency of the water due to seasonal variations in atmospheric temperature. In contrast, the stratification of meromictic lakes is mainly controlled by the ion content in the water column. Although there are less than 200 documented meromictic lakes worldwide, there may be more unmonitored examples (Gulati et al. 2017). Permanently stratified lakes are usually divided into three parts: (1) the lower, monimolimnion layer is anoxic and rich in dissolved ions with poor hydrodynamic circulation; (2) the upper, mixolimnion zone behaves essentially as a holomictic lake directly influenced by the meteorological forcings (mainly air temperature); and (3) the region in between, referred to as the chemocline, displays a steep chemical gradient with inversion of the redox potential. Owing to the lack of mixing, the permanent stratification leads to a stable anoxic environment in the monimolimnion. This provides an ideal setting for studying biogeochemical processes mediated by microorganisms, such as the sulfur and nitrogen cycles that typically occur in anaerobiosis (Kappler and Bryce 2017). In the few cases of meromictic lakes where light can penetrate the anoxic depths of the monimolimnion, dense bacterial layers (BLs) are formed and primarily composed of anoxygenic phototrophic sulfur bacteria (Overmann 2008; Diao et al. 2018; Wu et al. 2021; Lambrecht et al. 2021; Chen et al. 2021; Cohen et al. 2023; Cabello-Yeves et al. 2023; Lunina et al. 2023). Furthermore, these anoxygenic phototrophs play an essential role in the lake’s ecosystem, serving as an additional food source for the trophic web (Overmann et al. 1999; Camacho et al. 2001).

Lake Cadagno is a crenogenic meromictic lake characterized by deep, ion-rich, subaquatic inflows (Camacho et al. 2001; Otz et al. 2003) due to a peculiar geological conformation of the Piora Valley (Fig. S1). This formation comprises Triassic carbonate rocks, including tectonized dolomitic limestones, and gypsum deposits with Karstic hydrology. As a result, water flowing through the karst dolomite and resurfacing from sub-lacustrine springs in the southern region of the lake supplies high ionic content (9–10 mM; Ca^2+^, Mg^2+^, SO_4_^2−^ and HCO_3_^−^; Fig. S1) to the lower anoxic part (monimolimnion) of the water column (Del Don et al. 2001). The deep water of the monimolimnion remains isolated from the rest of the lake, resulting in the development of a stable anoxic environment that, according to the sediment records, dates to more than 10,000 years (Niemann et al. 2012; Ravasi et al. 2012; Wirth et al. 2013; Berg et al. 2022; Zander et al. 2023). The primary source of water for the mixolimnion is a small stream flowing from Lake Stabbio, a small lake located at 2351 m asl. The crystalline rocks (gneiss) in its catchment area are chemical-resistant, conferring the mixolimnion a low ionic strength. This leads to a disparity in density between the two water layers in Lake Cadagno, which in turns allows for the development of a highly stable stratification with a chemocline at a depth of approximately 10–12 m. This layer is distinguished by a rapid shift in the concentrations of chemical components, resulting in a redox stratification (Fritz and Bachofen 2000; Lüthy et al. 2000).

The high concentrations of sulfate (> 80 mg l^−1^) coming from the sub-lacustrine springs promoted the development of chemoheterotrophic anoxygenic sulfate-reducing bacteria (SRB). The metabolism of SRB leads to the production of hydrogen sulfide (H_2_S) in the deep layers of the lake (e.g., monimolimnion and sediment). Upon reaching the illuminated portion of the anoxic layer in the lower chemocline at approx. 12.0 m depth, H_2_S is used by anoxygenic phototrophic sulfur bacteria as an electron donor for anoxygenic photosynthesis (Frigaard and Dahl 2008). This leads to the development of a highly concentrated bacterial layer (BL; up to 10^7^ cell ml^−1^) composed of different species of purple (PSB) and green sulfur bacteria (GSB) (Fischer et al. 1996; Tonolla et al. 2005; Decristophoris et al. 2009; Gregersen et al. 2009; Danza et al. 2018; Saini et al. 2022).

One characteristic species is the PSB *Chromatium okenii* (Luedin et al. 2019), which accounts for most of the BL biovolume owing to its large cell size (approx. 10.0 µm main rod axis length). This microorganism is capable of active movement via a flagellar tuft, swimming toward light (positive phototaxis) and away from oxygen (negative aerotaxis). This results in an increase in water density in the chemocline zone, which “sinks” and drags the microorganisms down, triggering a phenomenon known as bioconvection (Sommer et al. 2017; Sepúlveda Steiner et al. 2019, 2021). The other PSB species present in the BL share a similar smaller spherical cell shape with a diameter of approx. 4.0 µm. The reference species is *Thiodictyon syntrophicum* (Peduzzi et al. 2012), which is the most abundant of the small-celled PSB, and has been previously studied for its ability to fix CO_2_ (Storelli et al. 2013, 2014) and to form aggregates with SRB (Peduzzi et al. 2003). The BL hosts four additional species of small-celled PSB (Tonolla et al. 2005). From a purely numerical perspective, both GSB, *Chlorobium phaeobacteroides* and *C. chlatratiforme*, represent the most abundant members of the BL. However, owing to their small size (less than 1.0 µm), they contribute only minimally to the total biovolume (Di Nezio et al. 2021). The remarkable biodiversity of anoxygenic phototrophic sulfur bacteria in the BL can be attributed to the implementation of different evolutionary strategies that enable their coexistence within the same ecological niche (Di Nezio et al. 2023). Previous studies reported a consistent and dynamic succession of the various BL populations. In particular, the motile PSB *C. okenii* exhibits a rapid growth during the summer months (June to August) and a decline phase in fall (September and October), which coincides with the development of the other, smaller PSB and GSB (Bosshard et al. 2000; Danza et al. 2018).

**Fig. 1:**
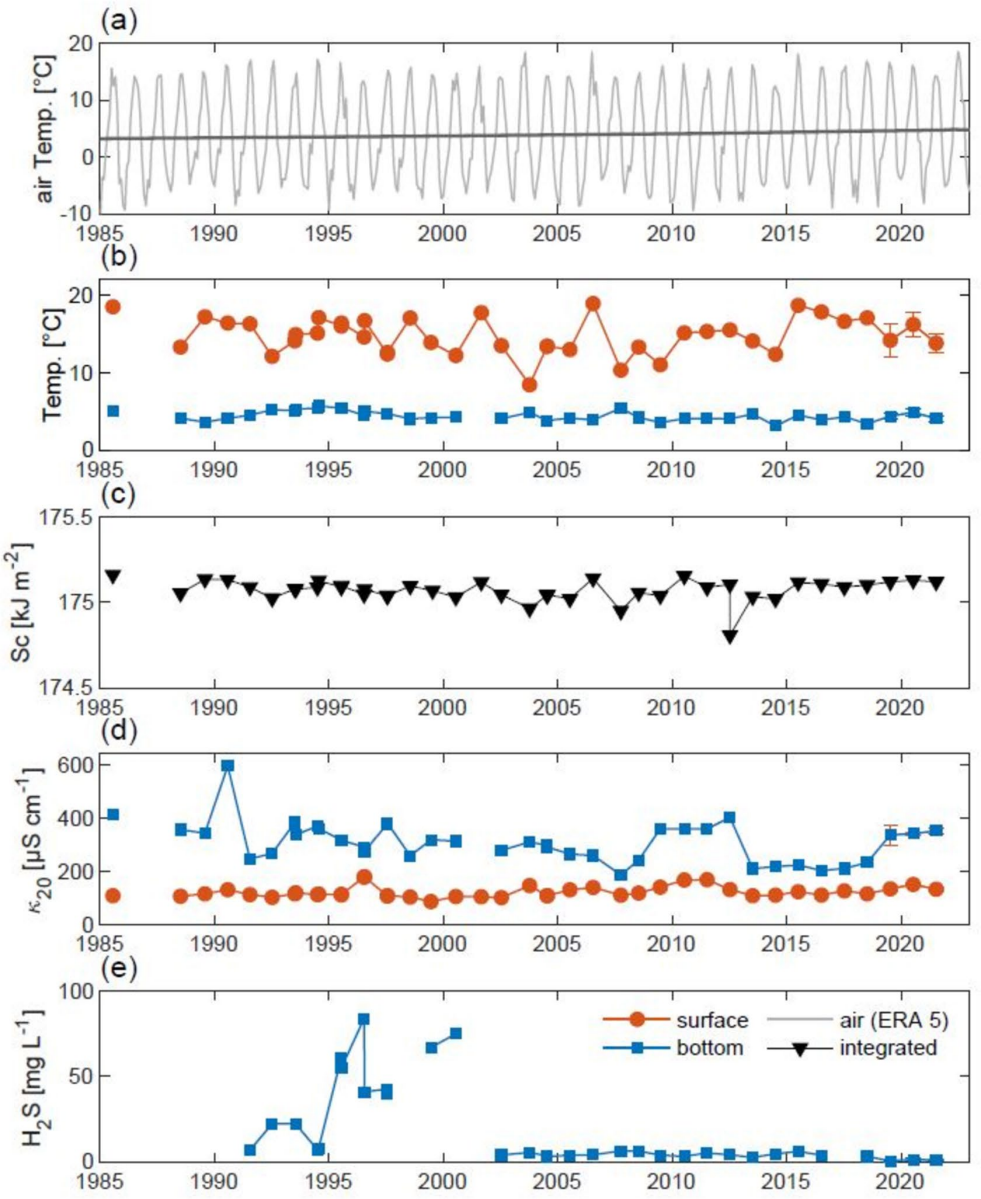
Historical trends in Lake Cadagno. **a** Air temperature obtained from ERA5-land weather reanalysis (light gray line) and its respective trend (~ 0.0325 °C year^−1^; dark-gray line). **b** Temperature (°C) of water at the surface (red dots) and bottom (blue squares). **c** Schmidt stability (Sc; kJ m^−2^) was obtained by processing multiparametric probe temperature and conductivity data following Eq. 2 (Fernández Castro et al. 2021). **d** Conductivity normalized to 20 °C (*κ*_20_; µS cm^−1^) at the surface (red dots) and bottom (blue squares). **e** Hydrogen sulfide (H_2_S; mg l^−1^) concentration at the lake bottom of the lake (between 18 and 20 m). Most measurements were taken in July from a platform placed in the limnetic zone during the undergraduate course in Microbial Hydrology, which began in 1985 (still offered) in the biology curriculum at the University of Geneva (UNIGE). Data from the years comprising this study (2019–2021) are included in the times series as arithmetic averages of the season-wide dataset accompanied by their respective standard deviations (error bars). The standard deviations of bottom temperature, *κ*_20_ and H_2_S, as well as Sc, are included, but the values are tiny and, therefore, not perceptible

The meromictic Lake Cadagno has been continuously monitored over the past 35 years (Fig. 1), producing multiple publications, related to different scientific area, such as its physical stratification (Del Don et al. 2001; Imboden 2013; Sepúlveda Steiner et al. 2019), its paleoproxy chemical composition (Canfield et al. 2010; Posth et al. 2017; Janssen et al. 2022), and its anoxygenic sulfur bacteria population (Egli et al. 1998; Musat et al. 2008; Philippi et al. 2021; Saini et al. 2022). In general, all these studies were conducted at specific times, limited to a few days during the warm season (June to October) when the lake was accessible. In addition, the monitoring and sampling were often influenced by meteorological conditions, with sunny days and optimal conditions being particularly well suited for these activities (Rand et al. 2022).

In this study, we conducted a comprehensive monitoring of the water column of Lake Cadagno throughout the entire warm season, spanning from June to October, over the course of three consecutive years (2019–2021). During the three years of the study, a range of physical, chemical, and biological parameters were monitored. These included temperature, conductivity, and light, as well as oxygen and sulfide levels, photosynthetic pigments, and turbidity. The resulting datasets were used to generate three high-frequency profiles, covering the period from June to October. The three profiles were then compared with one another and related to external weather conditions, with a particular focus on to the developmental trends of the main types of anoxygenic phototrophic sulfur bacteria present in the BL.

## Materials and methods

### Site description

Lake Cadagno is a crenogenic meromictic lake located in the Piora Valley at 1921 m a.s.l. in the southern Swiss Alps (Table 1).

**Table 1:** Morphometric date of meromictic Lake Cadagno 46° 33′ N, 8° 43′ E

Length (m)	842
Width (m)	423
Depth (m)	21.5
Surface (m^2^)	261,000
Volume (m^3^)	2,420,000

The surface area of Val Piora is 23.3 km^2^, including 5.3 km^2^ below 2000 m, 8.6 km^2^ between 2000 and 2200 m, and 9.4 km^2^ above 2200 m. The Piora Valley lies at the middle of a geologically complex area of the Alps (Fig. S1). Three well-defined massifs can be identified. In the northern sector, the Gotthard massif is composed of crystalline rocks originating from the pre-Mesozoic era. Subsequently, the Piora Valley is traversed by a syncline of sedimentary rocks dating back to the Triassic (approximately 230 million years ago). These rocks extend across the Gotthard paleo-massif. In the southern sector lies the Lukmanier Massif, separated from the Gotthard Massif and the sedimentary rocks of the Piora Valley. Composed of crystalline rocks, it is regarded as the front of the Lower Pennine nappes. The valley receives approximately 1400 mm of precipitation annually, which is relatively low for its altitude. One notable feature of Val Piora is the prevalence of limnic environments, including 21 lakes, 28 ponds, 15 marshes, and 58 streams. The crest line between the Pizzo Taneda and the Schenadüi represents the watershed between the Mediterranean and the North Sea.

### Sampling, limnological analyses, and climate data

Lake Cadagno is a crenogenic meromictic lake located in the Piora Valley at 1921 m a.s.l. in the southern Swiss Alps (46° 33′ N, 8° 43′ E, depth 21 m). All measurements were conducted from a platform placed in the limnetic zone. Until 2015, physicochemical parameters of the water column were measured using a YSI 6000 profiler (Yellow Springs, Inc., Yellow Springs, OH). These parameters included temperature (°C), conductivity (µS cm^−1^), pH, dissolved oxygen (mg l^−1^), redox potential (ORP; mV), and turbidity (FTU, formazine turbidity unit). Sampling was conducted using an 800-ml Niskin bottle attached to a second winch with samples collected at 1-m intervals in the mixolimnion and monimolimnion and every 0.2 m in the chemocline, as shown in Supplementary Fig. S2 and previous studies (Tonolla et al. 2003; Storelli et al. 2013). From 2016, parameters of the water column were determined using a multiparameter probe (CTD115M, Sea&Sun Technology, Germany) equipped with sensors measuring pressure (mBar), temperature (°C), conductivity (µS cm^−1^), dissolved oxygen (mg l^−1^), turbidity (FTU), Blue Green Algae Sensors: Phycocyanin (BGA-PC) and photosynthetic active radiations (PAR), in combination with a Tygon tube (20 m long, inner diameter 6.5 mm and volume 0.66 L) and a peristaltic pump (KNF Neuberger Inc., USA) for the BL sampling, as referenced (Di Nezio et al. 2021).

Turbidity served as a proxy for determining the position of the BL in the water column. Specifically, a consistent peak (> 10 FTU) in the turbidity profile was used as a physical signature of the BL and sampling depths were determined accordingly (see red graph in Supplementary Fig. S2). Water samples were collected 1 m above, at the top, 50 cm within, at the bottom, and 1 m below the BL. Samples were stored in 1.5-ml Eppendorf tubes for flow cytometry analysis and in 12.0-ml glass vials containing 4.0% zinc chloride (ZnCl_2_) solution to avoid oxidation of the sulfide. All samples were kept cold and dark before being analyzed within a few hours at the Alpine Biology Center (CBA) facilities directly in Piora. Chemical analysis for sulfide was completed with a colorimetric kit (Sulfide Test—114779, Merck, Switzerland) following the users’ manual and quantified by Spectroquant spectrophotometer (Pharo 300, Merck, Switzerland).

Atmospheric radiation data at 10-min resolution were retrieved from a meteorological station (istSOS; https://hydromet.supsi.ch/) close to the lake shore. This station is equipped with temperature and humidity sensors (Rotronic), a rainfall meter (1518 H3, Lambrecht), a pyranometer (CNR-4, Kipp&Zonen), and a weathervane-oriented anemometer (L14512, Lambrecht). Despite some technical issues in the first year of monitoring (2019, pyranometer), we collected regular air temperature, solar radiation, and rainfall data for three consecutive years (Fig. 4). The meteorological data used for analysis from the past 30 years was obtained from the Copernicus Climate Change Service (C3S) Climate Data Store (2017): ERA5, fifth generation of ECMWF atmospheric reanalyses of the global climate.

### Flow cytometry

A BD Accuri C6 cytometer (Becton Dickinson, San Jose, CA) equipped with two lasers (488 and 680 nm), two scatter detectors, and four fluorescence detectors (laser 488 nm: FL1 = 533/30, FL2 = 585/40, FL3 = 670; laser 640 nm: FL4 = 675/25) was used for analysis of samples. A threshold of 2000 on FSC-H was applied to exclude most of the unwanted abiotic particles. Furthermore, a FL3-A > 1100 threshold was applied to FL3 (red fluorescence) to discriminate cells emitting autofluorescence due to chlorophyll and bacteriochlorophyll. Phototrophic sulfur bacteria were enumerated by flow cytometry (FCM), measuring (bacterio)chlorophyll-like autofluorescence particle events as described by Danza et al. (2017 and 2018).

### Physical calculations

The water density (${\rho }_{w}$; without bacteria) was calculated using the ionic water composition of Lake Cadagno (Uhde 1992) as implemented in Sepúlveda Steiner et al. (2021), represented as:

1 $$\rho_{w} \left( {T,S} \right) = \rho_{w}^{\prime } \left( T \right) + \beta S,$$

where $\rho_{w}^{\prime } = 999.84\, + \,6.55 \times 10^{ - 2} \,{\text{T}}\, - \,8.56\, \times \,10^{ - 3} {\text{ T}}^{2} \, + { }5.94\, \times \,10^{ - 5} {\text{T}}^{3} { }$   is the temperature (T)-dependent water density and *β* = 0.96 × 10^−3^ kg g^−1^ is Lake Cadagno’s water haline contraction coefficient. Salinity (S) is obtained using the expression *S* =* ακ*_20_, where *α* = 0.72 × 10^−3^ kg m^−3^ (μS cm^−1^)^−1^ is the ion-specific conductivity to salinity factor for Lake Cadagno (Uhde 1992) and *κ*_20_ (μS cm^−1^) is conductivity normalized to 20 °C.

To quantify the overall water column stability, we calculated the Schmidt stability index (Sc) (Schmidt 1928; Idso 1973). This quantity represents the amount of mechanical work per unit area required to vertically mix the water column of a density-stratified lake. Using the CTD data, Sc was calculated as (Fernández Castro et al. 2021):

2 $$\mathrm{Sc}=\frac{1}{{A}_{o}}{\int }_{0}^{{z}_{bot}}g{\rho }_{w}\left(z-{z}_{v}\right)A\left(z\right) \mathrm{d}z,$$

where *A*_o_ = 0.23 km^2^ is the surface area, *g* = 9.81 m s^−2^ is the gravitational acceleration, *z*_bot_ is the lake bottom depth (21 m), *A*(*z*) is the lake’s hypsometric curve as a function of depth (*z*), and *z*_v_ = 5.3 m is the depth of the lake’s center of volume (Fernández Castro et al. 2021).

## Results

### Retrospective on the stratification stability of Lake Cadagno

The meromictic Lake Cadagno has been the subject of study for nearly four decades, allowing us to monitor the stability of the water column over several seasons (Fig. 1).

The trend line shows an increase in air temperature (Fig. 1a, dark-grey line) of approximately 0.0325 °C year^-1^, confirming the observed planetary climate change record of a progressive increase in global average temperatures of around 1.5–2.0 °C (ERA5-land weather reanalysis). When we look at the water temperature profiles (Fig. 1b), they appear to be uniform for both depth of the water column, surface (mixolimnion, red dots), and depth (monimolimnion, blue squares), different compared with the air (gray line). Surface water was on average 15.24 ± 2.13 °C, where the highest value was found in 2006 (July 18) with 18.91 °C while the lowest in 2021 with 11.5 °C (July 14). Conductivity (Fig. 1d) remains very stable at the surface (mixolimnion, red dots), while it has a slight change in depth (monimolimnion, blue squares), oscillating between about 400 and 220 µS cm^−1^ over the past 40 years, with a maximum peak of about 600 in 1990. Despite this, the stability of stratification remains almost unchanged with an average of 175.06 ± 0.07 kJ m^−2^ (Fig. 1c). Regarding sulfide measurements (Fig. 1e), an increase can be seen in the period between 1995 and 2000 with values rising from around 5.0 to over 75. 0 mg l^−1^, then returning to around 5.0 from 2001 to the present.

### Intensive monitoring of the water column (2019–2021)

**Fig. 2:**
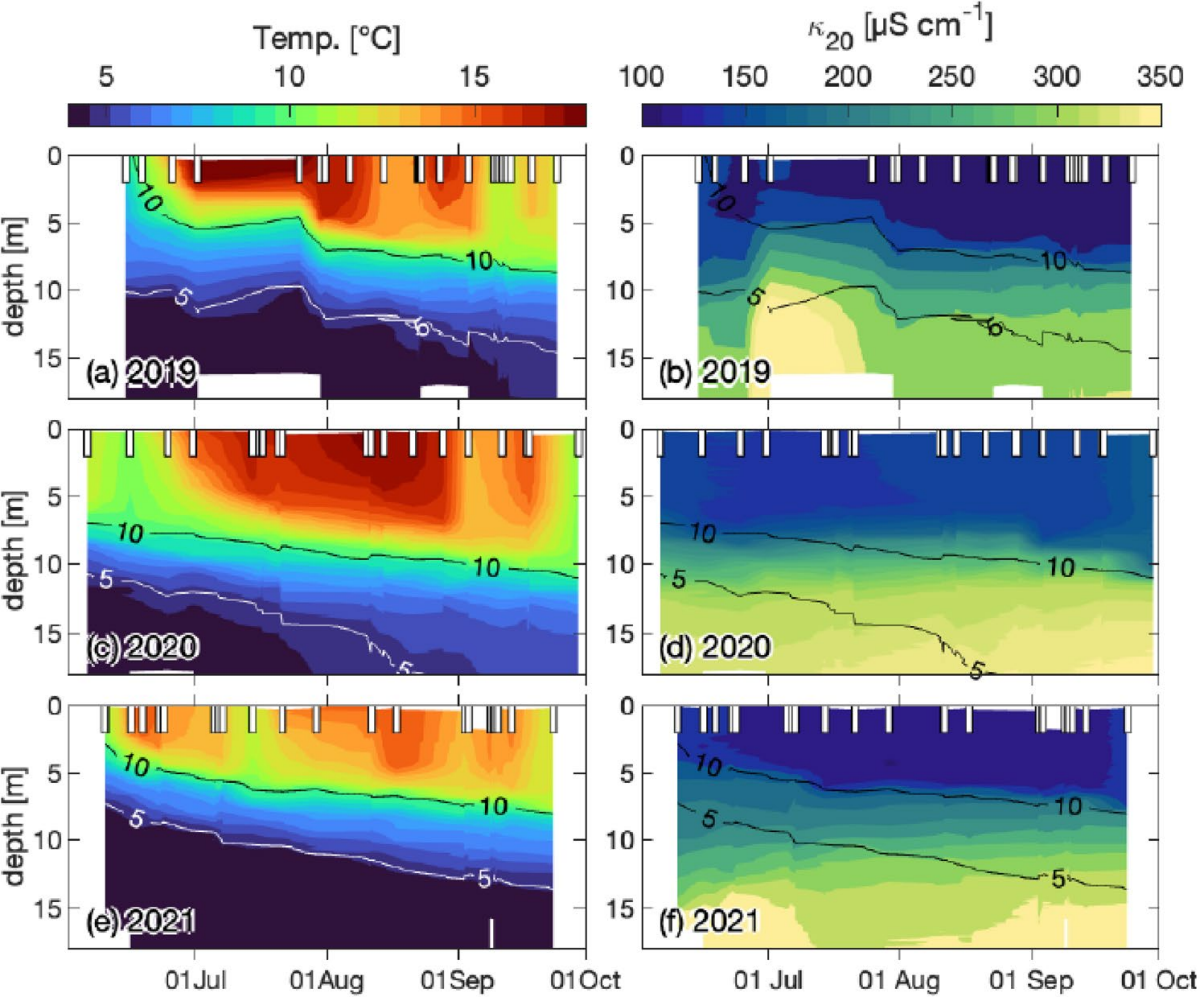
Water column profiles for years 2019, 2020, and 2021: physical properties. **a**, **c**, **e** Temperature (Temp.; °C). **b**, **d**, **f** Conductivity normalized to 20 °C (*κ*_20_; µS cm^−1^). The black and white continuous lines represent isothermal depths at 10 °C (highest line) and 5 °C (lowest line). White bars on the upper part of each plot indicate the sampling date

**Fig. 3:**
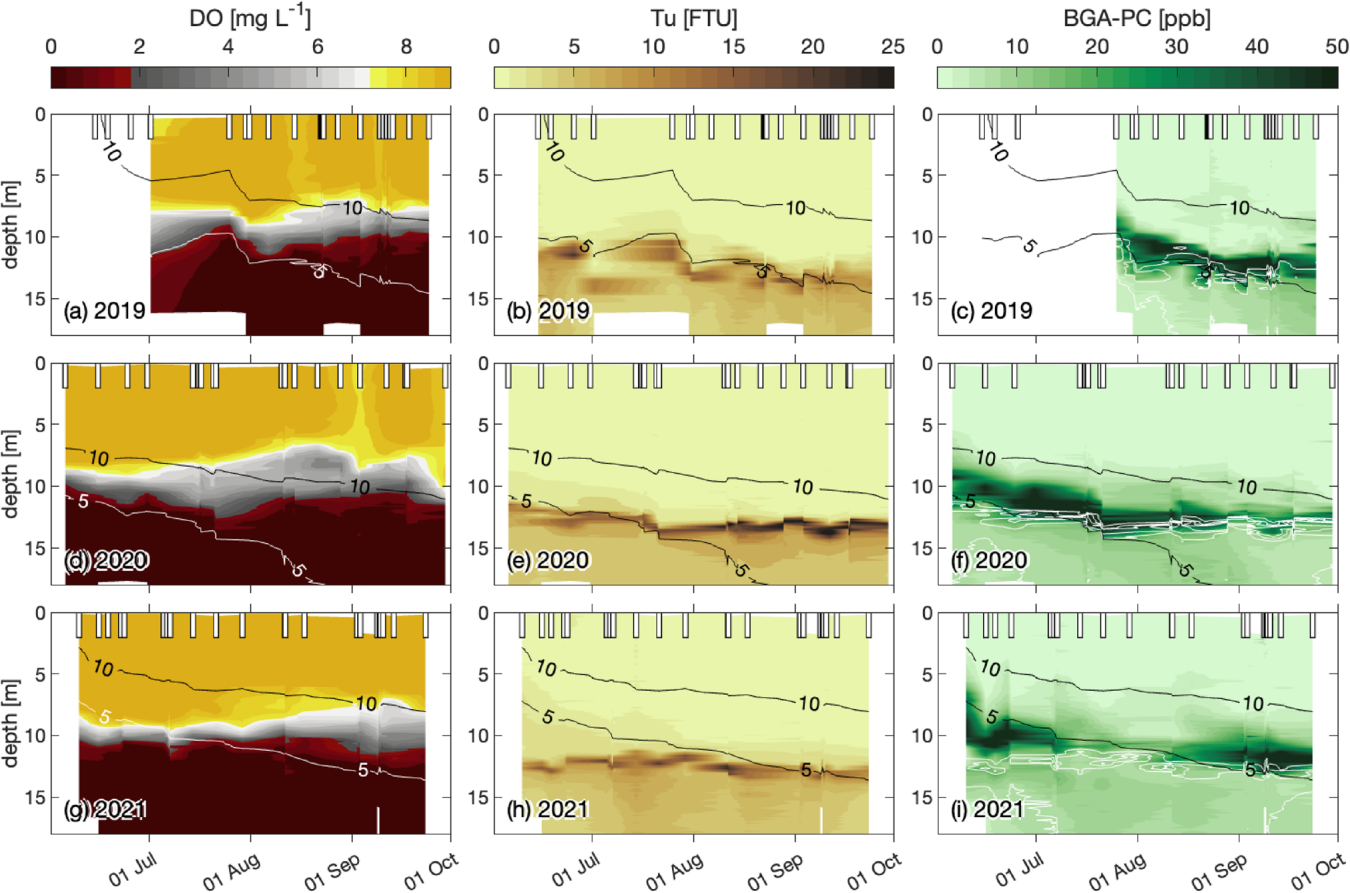
Water column profiles for years 2019, 2020, and 2021: biochemical properties. **a**, **d**, **g** Dissolved oxygen (DO; mg L^−1^). **b**, **e**, **h** Turbidity (Tu; FTU), an ad hoc proxy for the bacterial layer (BL). **c**, **f**, **i** Blue–green algae in terms of phycocyanin (BGA-PC; ppb). The black and white continuous lines tagged with 10 and 5 represent isothermal depths at 10 °C (highest line) and 5 °C (lowest line), respectively. White contours on BGA-PC graphs (**c**, **f**, **i**) depict the turbidity peak and show that blue–green was often concentrated above the BL. White bars on the upper part of each plot indicate the sampling date. The missing DO and BGA-PC profiles in 2019 are due to broken sensors at the time of the sampling

Lake Cadagno is typically accessible only during the warm season, from June to October, which is why our continuous measurements were made only at this time of year. The water column profiles measured in the warm season from 2019 to 2021 were divided as follows: Fig. 2 to show the stability of stratification through temperature and conductivity profiles, and Fig. 3 to show the parameters related to the localization of anoxigenic phototrophic sulfur bacteria using turbidity, oxygen and phycocyanin autofluorescence values.

The temperature and conductivity values exert a direct influence on the density of water, which in turn affects its stratification. The presence of two separate water layers appears clearly in all profiles measured during the study, the mixolimnion from 0 to about 10 m, which is subject to external environmental factors (e.g., wind and sun), and from about 10 m to the bottom the monimolimnion, which is completely isolated as shown by the constant temperature and conductivity profiles.

The chemocline, where the oxide–reductive transition occurs, is then at about 10 m depth, as evidenced by the oxygen profile, with a rapid reduction to a situation of complete absence, characteristic of the lower layer. Here we find the presence of two distinct populations of phototrophic microorganisms: in the upper part of the chemocline, the community of cyanobacterial-like microorganisms evidenced by the presence of their typical pigment (BGA-PC), and in the lower part, in the absence of oxygen and presence of sulfide, the large community of anoxygenic phototropic sulfur bacteria forming a characteristic bacterial layer (BL) with high turbidity values (> 10 FTU). Microbiological development shows some differences among the 3 years of our monitoring, in both concentration and location.

### Insight into microbiological turbidity profiles

**Fig. 4:**
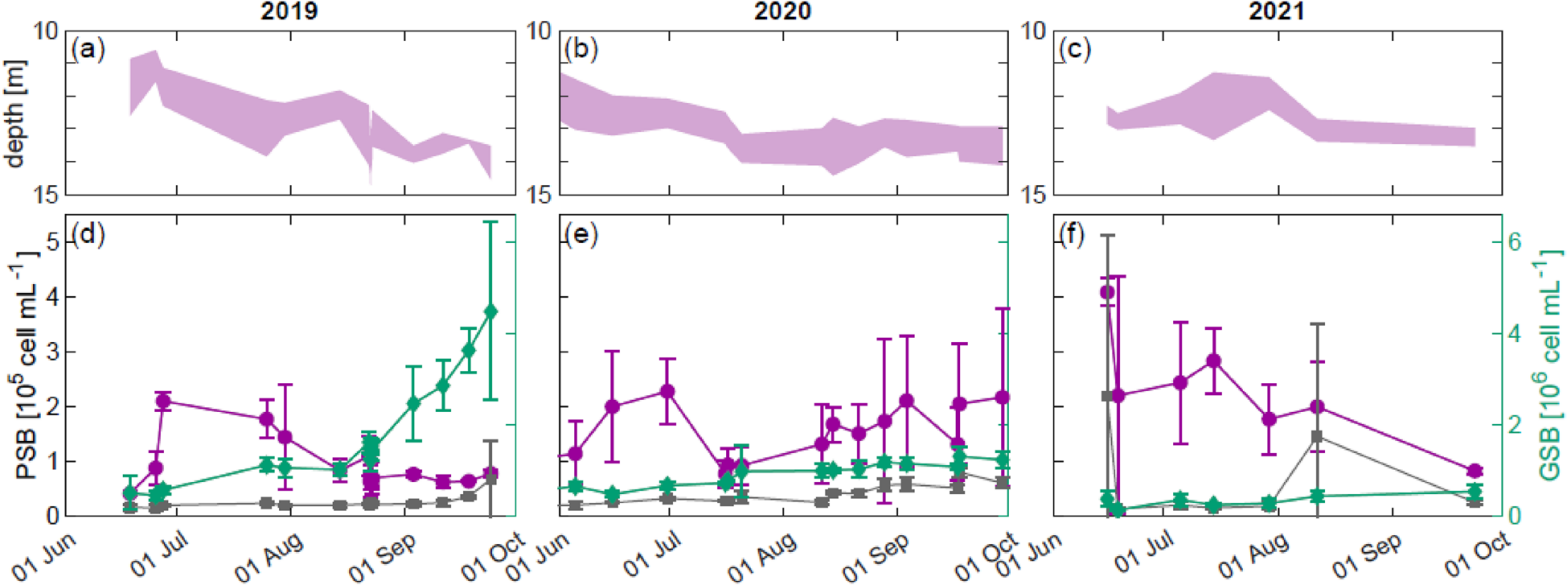
Monitoring of the BL and its anoxygenic phototrophic community. **a**–**c** BL extent (pink area) displaying thickness and depth for 2019–2021, the black line correspond to 13.5 m of depth. The BL was obtained from turbidity values > 10 FTU. **d**–**f** Bacteria cell counts in the BL for the three main phenotype of anoxygenic phototrophic sulfur bacteria: large cells PSB *C. okenii* (purple), the five species of small cells PSBs (gray), and both GSB species (green). The values presented correspond to arithmetic means and respective standard deviations (error bars) obtained by considering samples at the top, 50 cm, and bottom of the BL. Counts of the different microorganisms were performed with flow cytometry based on cell size and fluorescence

At each water column measurement, water samples were collected in the lower anoxic chemocline zone at the increase in turbidity profile (> 10 FTU, Fig. 3b, e, h), to monitor the seasonal evolution of different anoxygenic phototrophic sulfur bacteria populations in the BL (Fig. 4).

The thickness and depth of the BL exhibited distinct inter- and intra-warm-season trends (Fig. 4a–c). In 2019, we observed a constant lowering and reduction in thickness, similar to what has already been observed in other studies (Danza et al. 2018). Similarly, in 2020, the BL followed the same general pattern, except at the end of the warm season; in fact, in September, the BL rises slightly and maintains a large thickness, more typical of the early summer months. Finally, in 2021, we observe a difference in the first part of the warm season, in the summer months, with a BL that tends to raise its position instead of lowering. This maintains its position always above 13.5 m, although there is a process of lowering in the second part of the warm season.

The different profiles of the BL are related to the different developmental dynamics of the three most important phenotypes present in the BL, namely the large-cell PSB (*C. okenii*), the small-cell PSB (6 different species), and the two GSB (*Chlorobium chlatratiforme* and *phaeobacteroides*) (Di Nezio et al. 2023). In 2019, we observed an early growth of the large-cell PSB *C. okenii* at the beginning of the summer period, then stabilizing and decreasing from August onward. The opposite trend is observed for GSBs, which begin their growth at the time when we observe a decrease in *C. okenii* (in August). The small PSB cells seem to remain constant in number, with a slight increase at the end of the warm season (late September). In 2020, the same trend observed in the previous year reappears. However, in the second half of the year, a further growth peak of large-cell PSB *C. okenii* occurs between August and October, which inhibits the GSB growth observed the previous year. Similarly, the seasonal dynamics of small PSB remain unchanged, showing a constant presence with a slight growth at the end of the warm season. In 2021, the large PSB *C. okenii* exhibits the same pattern as in 2019, with a strong growth between June and July followed by a steady decline. However, this time, GSB do not show a significant growth phase and remain stable throughout the warm season. Unlike the previous two years, the small PSB experience slight growth in August but does not undergo a secondary increase in late September.

### Weather during the 3 years of monitoring

**Fig. 5:**
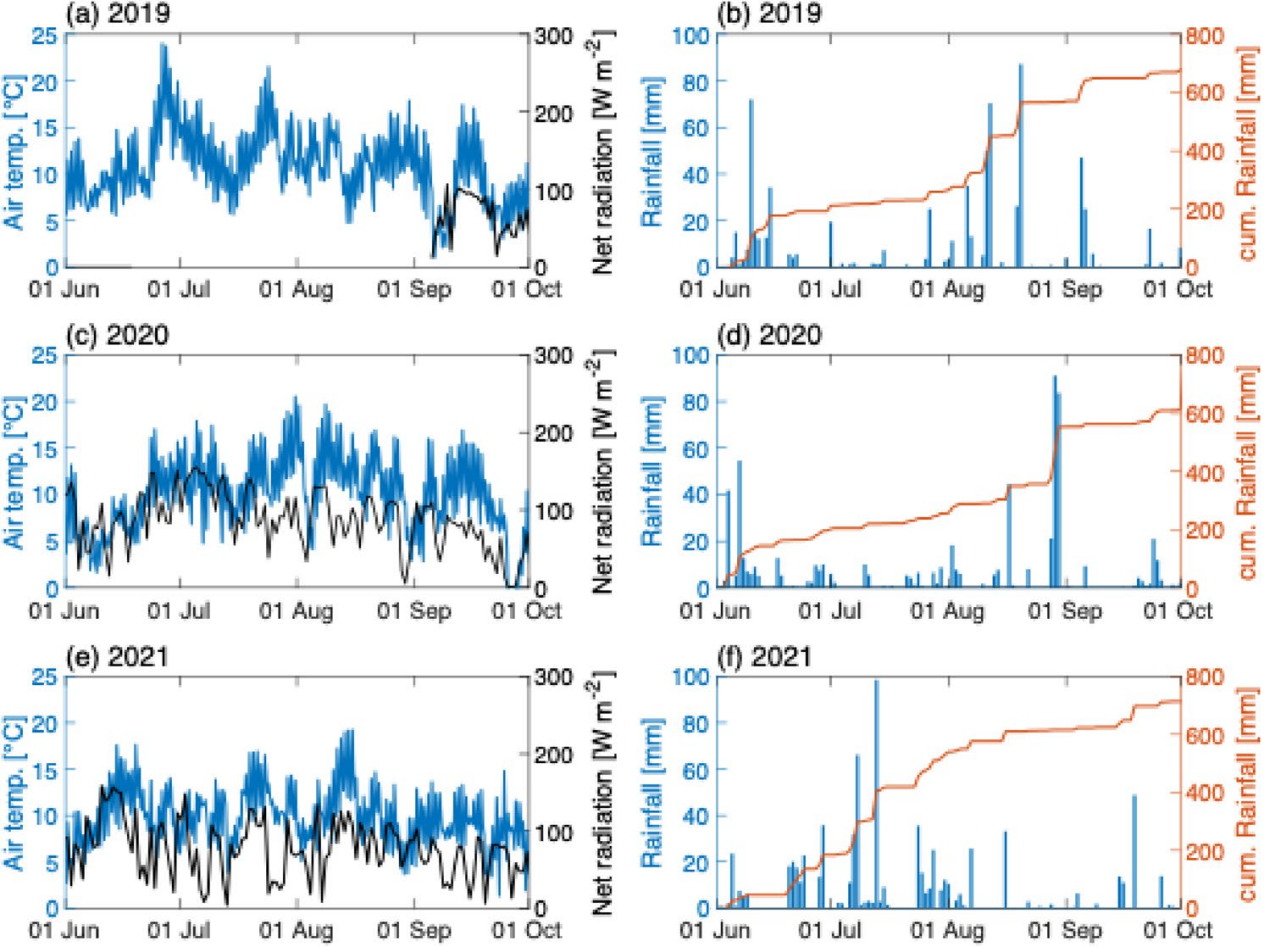
Meteorological data of the warm season for the three years of monitoring (2019–21). **a**, **c**, **d** 8-h averaged air temperature (°C; blue lines) and daily-averaged net radiation (W m^−2^; black lines) for 2019, 2020, and 2021. **b**, **d**, **f** Daily rainfall (mm; blue bars) accompanied by the cumulative rainfall (red lines) in the same period for 2019, 2020, and 2021, respectively

Weather readings were collected via a meteorological station near Lake Cadagno (< 100 m) at the Alpine Biology Center (CBA, coordinates 46° 54′ N, 8° 71′ E) foundation (Fig. 5).

The air temperature data indicate minimal variation between the three years considered (Fig. 5a, c, e, blue graph) when viewed in graphical form. However, when the data are examined on a monthly basis, considerable discrepancies are evident, with monthly averages differing by more than 1 °C. Indeed, with the exception of September, which exhibited three comparable average temperatures of approximately 8.29 ± 0.25 °C, notable discrepancies in the mean temperature were observed during the summer months across the three years under consideration. The month of June in 2019 was the hottest, with an average temperature of 11.16 ± 3.56 °C, followed by June 2021, which had an average temperature of 10.05 ± 2.48 °C. In contrast, June 2020 was significantly colder, with an average temperature of 8.07 ± 3.00 °C. The warmest month in Piora is July, with an average temperature of approximately 11.69 ± 1.01 °C. However, in 2021, the average temperature was about 1 °C lower at only 10.60 ± 1.99 °C.

The correlation between high temperatures and the presence of sunshine is well established; conversely, the incidence of rainfall is inversely proportional to such conditions, particularly during the summer months. The data for July reveal a clear explanation for the low average temperatures observed in 2021, with precipitation levels exceeding those of the corresponding months in 2019 and 2020 by a factor of 5 or more. The exceptional precipitation in July 2021 also resulted in a reduction in the availability of solar energy, with net radiation levels being one-third lower than those observed in 2020. Net radiation measurements were not feasible in 2019 owing to the malfunction of the pyranometer. Regarding the total precipitation, it is noteworthy that the situation appears to be reversed in August, with considerably more precipitation in 2019 and 2020 than in 2021.

## Discussion

This study provides a comprehensive description of the warm season dynamics of the community of anoxygenic phototrophic sulfur bacteria community within the bacterial layer (BL). This community of primordial phototrophs is a distinctive feature of the meromictic Lake Cadagno, which, owing to its permanent stratification, can host environments in its depths that are possible proxies to understand the past oceans. A three-year monitoring program, including physicochemical and meteorological parameters within the water column, showed variations throughout the monitoring period in the anoxygenic phototroph populations within the BL, despite the constant stability of stratification. In particular, the populations of large-cell PSB *C. okenii*, small-cell PSB, and both GSB exhibited different growth dynamics over the three years of fine-scale monitoring, indicating the impact of external meteorological factors, such as precipitation and, conversely, light irradiance.

The development of BL, along with other anoxygenic microorganisms in the monimolimnion, depends on maintaining the meromictic nature of the lake. A key question is thereby to assess how external factors can influence the stability of the meromixis and the structure of the lake. One way to estimate the stability of the water column of a lake is through the Schmidt stability index, which determines the energy required to fully mix the water column (Schmidt 1928; Idso 1973). Over the course of the three-year monitoring period, a highly stable stratification was observed with a value that remained constant at 175 ± 0.02 kJ m^−2^. This is approximately ten times higher than the value observed in lakes of a similar size (Borics et al. 2015).

Moreover, the stability of the stratification is corroborated by data collected from 1985 onward in July (14B641—Microbial Hydrology course at the University of Geneva). At the chemical level, a notable increase in sulfide concentration was observed between 1995 and 2000 (Fig. 1e). The underlying cause of this phenomenon remains uncertain, but it may have facilitated the emergence of the species *C. chlatratiforme*, which appeared in 1999 (Tonolla et al. 2005; Decristophoris et al. 2009; Gregersen et al. 2009). However, the stability of the meromictic Lake Cadagno has not been affected in a chemical sense by rising temperatures in the context of the ongoing process of climate change, particularly in recent years (Kraemer et al. 2021; Gilarranz et al. 2022). Historical evidence spanning more than 10,000 years, analyzed through sediment studies (Wirth et al. 2013; Berg et al. 2022; Zander et al. 2023), suggests the persistence of physical stratification and anoxygenic phototrophic sulfur bacteria community dynamics (Ravasi et al. 2012).

Provided that the two-layer structure is maintained, this lake will continue to serve as a natural laboratory for the study of anoxic lifeforms. Typically, PSB and GSB communities depend strongly on euxinic conditions and light availability. It is widely acknowledged that variations in light intensity exert a profound influence on the ecology of anoxygenic phototrophs. Indeed, GSB have been demonstrated to exhibit a competitive advantage over PSB in low-intensity environments, a phenomenon attributed to their superior antenna system, called chlorosomes (Bryant and Frigaard 2006). An interesting aspect, previously reported by Musat et al. (2008) and Danza et al. (2018), pertains to the seasonal succession of microbial communities. The BL in the chemocline shows a pronounced dominance of large-cell PSB *C. okenii* during the summer months, followed by a growth of GSB in the autumn. Indeed, in 2019, the study observed an early growth of *C. okenii* from June to August, with a subsequent decrease, and a simultaneous increase in GSB from August to October. Moreover, during July, large-cell PSB *C. okenii* produced an additional mixing process in the BL—bioconvection (Sommer et al. 2017; Sepúlveda Steiner et al. 2019, 2021). The resulting (biogenic) turbulent mixing also displaces small cells of PSB and GSB out of the “anoxygenic photosynthetic zone” (Di Nezio et al. 2023). These cells are incapable of resisting the transport caused by the stirring as they move passively through gas vacuoles (Overmann 2006, 2008). It is therefore evident that meteorological conditions are of paramount importance to the ecological dynamics of the BL anoxygenic phototrophic sulfur bacteria community, as they are the primary determinant of the amount of light available for their survival.

Owing to its geographical position in the Alps at 1921 m a.s.l., Lake Cadagno is covered with ice for almost half the year. In an average year, the lake freezes in early December and remains covered until mid-May, for about 5–6 months. Consequently, the sampling timeframe was constrained to the period between June and October. Access to the Piora Valley is only feasible during periods of snow-free conditions, precluding the possibility to extend the sampling period into November or December. During the three-year monitoring period (2019–2021), the warm season (June to October) was characterized by three distinct weather patterns. This gave us the opportunity to observe the effect of various external abiotic factors on the physicochemical and microbiological stability of Lake Cadagno. While no differences were observed in the stability of stratification (Fig. 2), the same cannot be said for the development of the BL’s anoxygenic phototrophic sulfur bacteria community. In fact, we observed three distinct warm season dynamics (Figs. 3, 4) with notable differences in the population developments of large-cell PSB *C. okenii* (especially in early summer) compared with small-cell PSB and GSB (especially in late summer). These dynamics were linked to precipitation (July 2021) and/or extremely strong storm events (late August 2020, Supplementary Fig. S3). In both exceptional weather situations, differences in the amount of light were observed in the presence of the BL’s anoxygenic phototrophic sulfur bacteria community (Fig. 5 or Table 2).

**Table 2:** Monthly sum of rainfall (mm), net radiation (J m^−2^), and wind speed (m s^−1^) for the 3 years of monitoring

	2019		2020		2021	
	Mean	St. dev.	Mean	St. dev.	Mean	St. dev.
Monthly mean temperature (°C)						
June	11.16	3.56	8.07	3.00	10.05	2.48
July	12.61	2.30	11.85	2.10	10.60	1.99
August	11.59	1.63	11.65	2.89	10.07	2.70
September	8.09	2.99	8.20	4.15	8.57	1.58
Monthly integrated net radiation (J m^−2^)						
June	N/A	N/A	2.33 × 10^8^	3.14 × 10^6^	2.37 × 10^8^	3.63 × 10^6^
July	N/A	N/A	2.96 × 10^8^	2.64 × 10^6^	1.93 × 10^8^	3.52 × 10^6^
August	N/A	N/A	2.28 × 10^8^	2.87 × 10^6^	2.25 × 10^8^	2.57 × 10^6^
September	N/A	N/A	1.57 × 10^8^	2.83 × 10^6^	1.55 × 10^8^	2.23 × 10^6^
Monthly total rainfall (mm)						
June	192.20	14.45	196.00	12.15	184.20	9.61
July	69.20	5.58	59.20	2.96	345.60	21.58
August	305.20	21.82	297.20	22.67	85.80	7.52
September	103.00	9.85	57.20	4.53	98.20	9.47

*N/A* not applicable

## Conclusions

Lake Cadagno represents a model ecosystem for studying the intricate interrelationships between physical, chemical, and microbiological stratification within an aquatic environment. Its permanent stratification and the presence of a distinctive anoxygenic microbial community provide unparalleled opportunities to investigate biogeochemical cycles and understand how external environmental factors impact anoxygenic biodiversity and productivity. Redox-stratified environments, such as euxinic or ferruginous systems (Swanner et al. 2020; Janssen et al. 2022), are fundamental for implementing knowledge of the primordial oceans of the Proterozoic era starting 2.5 billion years ago (Poulton et al. 2004; Scott et al. 2011).

In this study, we have shown that the stability of the meromixis of the lake remained unchanged during the three years of monitoring (2019–2021), with a well-defined chemocline around a depth of 10–12 m. At this depth, there is a marked reduction in oxygen and an increase in hydrogen sulfide, which creates ideal conditions for the development of a dense BL composed of anoxygenic phototrophic sulfur bacteria. Historical data collected since 1985 show that the stability of the stratification has not changed, despite the average rise in global temperatures. Despite the general stability of the lake’s stratification, the study revealed considerable variations in the composition of the BL microbial community among the three years of monitoring. Differences in growth patterns, e.g., of large-cell *C. okenii* and GSB, as well as changes in BL depth, and thickness, suggest that climate variability could affect microbial community structure and ecosystem functioning. These variations demonstrate the importance of long-term monitoring to capture interannual dynamics and ecosystem responses to climate change.

## Supplementary Information

Below is the link to the electronic supplementary material.

Supplementary file1 (DOCX 1028 KB)

## Data Availability

All raw data and metadata used for figure making are accessible through Zenodo (10.5281/zenodo.10663093).

## References

[CR1] Berg JS, Lepine M, Laymand E et al (2022) Ancient and modern geochemical signatures in the 13,500-year sedimentary record of Lake Cadagno. Front Earth Sci (Lausanne) 9:754888. 10.3389/FEART.2021.754888/BIBTEX

[CR2] Boehrer B, Schultze M (2008) Stratification of lakes. Rev Geophys 46:2005. 10.1029/2006RG000210

[CR3] Borics G, Abonyi A, Várbíró G et al (2015) Lake stratification in the Carpathian basin and its interesting biological consequences. Inland Waters 5:173–186. 10.5268/IW-5.2.702

[CR4] Bosshard PP, Santini Y, Grüter D et al (2000) Bacterial diversity and community composition in the chemocline of the meromictic alpine Lake Cadagno as revealed by 16S rDNA analysis. FEMS Microbiol Ecol 31:173–182 10640670 10.1111/j.1574-6941.2000.tb00682.x

[CR5] Bryant DA, Frigaard NU (2006) Prokaryotic photosynthesis and phototrophy illuminated. Trends Microbiol 14:488–496. 10.1016/j.tim.2006.09.001 16997562 10.1016/j.tim.2006.09.001

[CR6] Cabello-Yeves PJ, Picazo A, Roda-Garcia JJ et al (2023) Vertical niche occupation and potential metabolic interplay of microbial consortia in a deeply stratified meromictic model lake. Limnol Oceanogr 68:2492–2511. 10.1002/LNO.12437

[CR7] Camacho A, Erez J, Chicote A et al (2001) Microbial microstratification, inorganic carbon photoassimilation and dark carbon fixation at the chemocline of the meromictic Lake Cadagno (Switzerland) and its relevance to the food web. Aquat Sci 63:91–106

[CR8] Canfield DE, Farquhar J, Zerkle AL (2010) High isotope fractionations during sulfate reduction in a low-sulfate euxinic ocean analog. Geology 38:415–418. 10.1130/G30723.1

[CR9] Chen YH, Chiang PW, Rogozin DY et al (2021) Salvaging high-quality genomes of microbial species from a meromictic lake using a hybrid sequencing approach. Commun Biol 4:1–12. 10.1038/s42003-021-02510-6 34426638 10.1038/s42003-021-02510-6 PMC8382752

[CR10] Cohen AB, Klepac-Ceraj V, Bidas K et al (2023) Deep photoautotrophic prokaryotes contribute substantially to carbon dynamics in oxygen-deficient waters in a permanently redox-stratified freshwater lake. Limnol Oceanogr 68:232–247. 10.1002/LNO.12262

[CR11] Danza F, Storelli N, Roman S et al (2017) Dynamic cellular complexity of anoxygenic phototrophic sulfur bacteria in the chemocline of meromictic Lake Cadagno. PLoS ONE 12:e0189510. 10.1371/journal.pone.0189510 29245157 10.1371/journal.pone.0189510 PMC5731995

[CR12] Danza F, Ravasi D, Storelli N et al (2018) Bacterial diversity in the water column of meromictic Lake Cadagno and evidence for seasonal dynamics. PLoS ONE 13:e0209743. 10.1371/journal.pone.0209743 30586464 10.1371/journal.pone.0209743 PMC6306205

[CR13] Decristophoris P, Peduzzi S, Ruggeri-Bernardi N et al (2009) Fine scale analysis of shifts in bacterial community structure in the chemocline of meromictic Lake Cadagno, Switzerland. J Limnol 68:16–24

[CR14] Del Don C, Hanselmann KW, Peduzzi R, Bachofen R (2001) The meromictic alpine Lake Cadagno: orographical and biogeochemical description. Aquat Sci 63:70–90

[CR15] Di Nezio F, Beney C, Roman S et al (2021) Anoxygenic photo- and chemo-synthesis of phototrophic sulfur bacteria from an alpine meromictic lake. FEMS Microbiol Ecol. 10.1093/FEMSEC/FIAB010 33512460 10.1093/femsec/fiab010 PMC7947596

[CR16] Di Nezio F, Roman S, Buetti-Dinh A et al (2023) Motile bacteria leverage bioconvection for eco-physiological benefits in a natural aquatic environment. Front Microbiol 14:1253009. 10.3389/FMICB.2023.1253009 38163082 10.3389/fmicb.2023.1253009 PMC10756677

[CR17] Diao M, Huisman J, Muyzer G (2018) Spatio-temporal dynamics of sulfur bacteria during oxic–anoxic regime shifts in a seasonally stratified lake. FEMS Microbiol Ecol. 10.1093/FEMSEC/FIY040 29528404 10.1093/femsec/fiy040 PMC5939864

[CR18] Egli K, Wiggli M, Klug J, Bachofen R (1998) Spatial and temporal dynamics of the cell density in a plume of phototrophic microorganisms in their natural environment. Doc Ist Ital Idrobiol 121–126

[CR19] Fernández Castro B, Bouffard D, Troy C et al (2021) Seasonality modulates wind-driven mixing pathways in a large lake. Commun Earth Environ 2:1–11. 10.1038/s43247-021-00288-3

[CR20] Fischer C, Wiggli M, Schanz F et al (1996) Light environment and synthesis of bacteriochlorophyll by populations of Chromatium okenii under natural environmental conditions. FEMS Microbiol Ecol 21:1–9

[CR21] Frigaard NU, Dahl C (2008) Sulfur metabolism in phototrophic sulfur bacteria. Adv Microb Physiol 54:103–200 10.1016/S0065-2911(08)00002-7 18929068

[CR22] Fritz M, Bachofen R (2000) Volatile organic sulfur compounds in a meromictic alpine Lake. Acta Hydrochim Hydrobiol. 10.1002/1521-401X(20004)28:4%3c185::AID-AHEH185%3e3.0.CO;2-V

[CR23] Gilarranz LJ, Narwani A, Odermatt D et al (2022) Regime shifts, trends, and variability of lake productivity at a global scale. Proc Natl Acad Sci USA 119:e2116413119. 10.1073/PNAS.2116413119/SUPPL_FILE/PNAS.2116413119.SAPP.PDF 35994657 10.1073/pnas.2116413119 PMC9436327

[CR24] Gregersen LH, Habicht KS, Peduzzi S et al (2009) Dominance of a clonal green sulfur bacterial population in a stratified lake. FEMS Microbiol Ecol 70:30–41. 10.1111/j.1574-6941.2009.00737.x 19656193 10.1111/j.1574-6941.2009.00737.x

[CR25] Gulati RD, Zadereev ES, Degermendzhi AG (2017) Ecology of meromictic lakes, Ecogical studies. Springer, Cham

[CR26] Idso SB (1973) On the concept of lake stability. Limnol Oceanogr 18:681–683. 10.4319/LO.1973.18.4.0681

[CR27] Imboden DM (2013) The influence of biogeochemical processes on the physics of lakes. In: Imberger Jörg (ed) Physical Processes in Lakes and Oceans, 19 March 2013. American Geophysical Union (AGU), pp 591–612. 10.1029/CE054P0591

[CR28] Janssen DJ, Rickli J, Wille M et al (2022) Chromium cycling in redox-stratified basins challenges δ53Cr paleoredox proxy applications. Geophys Res Lett 49:e2022GL099154. 10.1029/2022GL099154 36589775 10.1029/2022GL099154 PMC9787902

[CR29] Kappler A, Bryce C (2017) Cryptic biogeochemical cycles: unravelling hidden redox reactions. Environ Microbiol 19:842–846. 10.1111/1462-2920.13687 28168803 10.1111/1462-2920.13687

[CR30] Kraemer BM, Pilla RM, Woolway RI et al (2021) Climate change drives widespread shifts in lake thermal habitat. Nat Clim Change 11:521–529. 10.1038/s41558-021-01060-3

[CR31] Lambrecht N, Stevenson Z, Sheik CS et al (2021) “Candidatus Chlorobium masyuteum”, a novel photoferrotrophic green sulfur bacterium enriched from a ferruginous Meromictic lake. Front Microbiol 12:1768. 10.3389/FMICB.2021.695260/BIBTEX 10.3389/fmicb.2021.695260 PMC8302410 34305861

[CR32] Luedin SM, Liechti N, Cox RP et al (2019) Draft genome sequence of chromatium okenii isolated from the stratified Alpine lake Cadagno. Sci Rep 9:1936. 10.1038/s41598-018-38202-1 30760771 10.1038/s41598-018-38202-1 PMC6374484

[CR33] Lunina ON, Grouzdev DS, Patsaeva SV et al (2023) Anoxygenic phototrophic bacteria of the Meromictic Lake Bol’shie Khruslomeny (Oleniy Island, Kandalaksha Gulf, Murmansk Oblast, Russia). Microbiology (Russian Federation) 92:792–806. 10.1134/S0026261723602051/FIGURES/11

[CR34] Lüthy L, Fritz M, Bachofen R (2000) In situ determination of sulfide turnover rates in a meromictic alpine lake. Appl Environ Microbiol 66:712–717 10653740 10.1128/aem.66.2.712-717.2000 PMC91885

[CR35] Musat N, Halm H, Winterholler B et al (2008) A single-cell view on the ecophysiology of anaerobic phototrophic bacteria. Proc Natl Acad Sci USA 105:17861–17866. 10.1073/pnas.0809329105 19004766 10.1073/pnas.0809329105 PMC2582579

[CR36] Niemann H, Stadnitskaia A, Wirth SB et al (2012) Bacterial GDGTs in Holocene sediments and catchment soils of a high Alpine lake: application of the MBT/CBT-paleothermometer. Climate past 8:889–906. 10.5194/CP-8-889-2012

[CR37] Otz MH, Otz HK, Otz I, Siegel DI (2003) Surface water/groundwater interaction in the Piora Aquifer, Switzerland: evidence from dye tracing tests. Hydrogeol J 11:228–239. 10.1007/S10040-002-0237-1/FIGURES/9

[CR38] Overmann J (2006) The family chlorobiaceae. The prokaryotes. Springer, New York, pp 359–378

[CR39] Overmann J (2008) Ecology of phototrophic sulfur bacteria. Sulfur metabolism in phototrophic organisms. Springer, Netherlands, pp 375–396

[CR40] Overmann J, Hall KJ, Northcote TG, Beatty JT (1999) Grazing of the copepod Diaptomus connexus on purple sulphur bacteria in a meromictic salt lake. Environ Microbiol 1:213–221. 10.1046/J.1462-2920.1999.00026.X 11207740 10.1046/j.1462-2920.1999.00026.x

[CR41] Peduzzi S, Tonolla M, Hahn D (2003) Isolation and characterization of aggregate-forming sulfate-reducing and purple sulfur bacteria from the chemocline of meromictic Lake Cadagno, Switzerland. FEMS Microbiol Ecol 45:29–37. 10.1016/S0168-6496(03)00107-7 19719604 10.1016/S0168-6496(03)00107-7

[CR42] Peduzzi S, Storelli N, Welsh A et al (2012) Candidatus “Thiodictyon syntrophicum”, sp. Nov., a new purple sulfur bacterium isolated from the chemocline of Lake Cadagno forming aggregates and specific associations with Desulfocapsa sp. Syst Appl Microbiol. 10.1016/j.syapm.2012.01.001 22386960 10.1016/j.syapm.2012.01.001

[CR43] Philippi M, Kitzinger K, Berg JS et al (2021) Purple sulfur bacteria fix N2 via molybdenum-nitrogenase in a low molybdenum Proterozoic ocean analogue. Nat Commun 12:1–12. 10.1038/s41467-021-25000-z 34362886 10.1038/s41467-021-25000-z PMC8346585

[CR44] Posth NR, Bristow LA, Cox RP et al (2017) Carbon isotope fractionation by anoxygenic phototrophic bacteria in euxinic Lake Cadagno. Geobiology. 10.1111/gbi.12254 28866873 10.1111/gbi.12254

[CR45] Poulton SW, Fralick PW, Canfield DE (2004) The transition to a sulphidic ocean 1.84 billion years ago. Nature 431:173–177. 10.1038/nature02912 15356628 10.1038/nature02912

[CR46] Rand JM, Nanko MO, Lykkegaard MB et al (2022) The human factor: weather bias in manual lake water quality monitoring. Limnol Oceanogr Methods 20:288–303. 10.1002/LOM3.10488

[CR47] Ravasi DF, Peduzzi S, Guidi V et al (2012) Development of a real-time PCR method for the detection of fossil 16S rDNA fragments of phototrophic sulfur bacteria in the sediments of Lake Cadagno. Geobiology 10:196–204. 10.1111/j.1472-4669.2012.00326.x 22433067 10.1111/j.1472-4669.2012.00326.x

[CR48] Saini JS, Hassler C, Cable R et al (2022) Bacterial, phytoplankton, and viral distributions and their biogeochemical contexts in Meromictic Lake Cadagno offer insights into the proterozoic ocean microbial loop. Mbio. 10.1128/MBIO.00052-22/SUPPL_FILE/MBIO.00052-22-S0002.PDF 35726916 10.1128/mbio.00052-22 PMC9426590

[CR49] Schmidt W (1928) Über die Temperatur- und Stabilitätsverhältnisse von Seen. Geogr Ann 10:145. 10.2307/519789

[CR50] Scott CT, Bekker A, Reinhard CT et al (2011) Late Archean euxinic conditions before the rise of atmospheric oxygen. Geology 39:119–122. 10.1130/G31571.1

[CR51] Sepúlveda Steiner O, Bouffard D, Wüest A (2019) Convection-diffusion competition within mixed layers of stratified natural waters. Geophys Res Lett 46:13199–13208. 10.1029/2019GL085361

[CR52] Sepúlveda Steiner O, Bouffard D, Wüest A (2021) Persistence of bioconvection-induced mixed layers in a stratified lake. Limnol Oceanogr 66:1531–1547. 10.1002/LNO.11702

[CR53] Sommer T, Danza F, Berg J et al (2017) Bacteria-induced mixing in natural waters. Geophys Res Lett. 10.1002/2017GL074868

[CR54] Storelli N, Peduzzi S, Saad MM et al (2013) CO₂ assimilation in the chemocline of Lake Cadagno is dominated by a few types of phototrophic purple sulfur bacteria. FEMS Microbiol Ecol 84:421–432. 10.1111/1574-6941.12074 23330958 10.1111/1574-6941.12074

[CR55] Storelli N, Saad MM, Frigaard N-U et al (2014) Proteomic analysis of the purple sulfur bacterium Candidatus “Thiodictyon syntrophicum” strain Cad16T isolated from Lake Cadagno. EuPA Open Proteom 2:17–30. 10.1016/j.euprot.2013.11.010

[CR56] Swanner ED, Lambrecht N, Wittkop C et al (2020) The biogeochemistry of ferruginous lakes and past ferruginous oceans. Earth Sci Rev 211:103430. 10.1016/J.EARSCIREV.2020.103430

[CR57] Tonolla M, Tonolla M, Peduzzi S et al (2003) Spatio-temporal distribution of phototrophic sulfur bacteria in the chemocline of meromictic Lake Cadagno (Switzerland). FEMS Microbiol Ecol 43:89–98. 10.1111/j.1574-6941.2003.tb01048.x 19719699 10.1111/j.1574-6941.2003.tb01048.x

[CR58] Tonolla M, Peduzzi R, Hahn D (2005) Long-term population dynamics of phototrophic sulfur bacteria in the chemocline of Lake Cadagno, Switzerland. Appl Environ Microbiol 71:3544–3550. 10.1128/AEM.71.7.3544-3550.2005 16000760 10.1128/AEM.71.7.3544-3550.2005 PMC1169024

[CR59] Uhde M (1992) Mischungsprozesse im hypolimnion des meromiktischen Lago di Cadagno: eine untersuchung mit hilfe natürlicher und künstlicher tracer. Unpublished MSc Thesis, Albert Ludwigs Universität Freiburg

[CR60] Wirth SB, Gilli A, Niemann H et al (2013) Combining sedimentological, trace metal (Mn, Mo) and molecular evidence for reconstructing past water-column redox conditions: the example of meromictic Lake Cadagno (Swiss Alps). Geochim Cosmochim Acta 120:220–238. 10.1016/j.gca.2013.06.017

[CR61] Wu YT, Chiang PW, Tandon K et al (2021) Single-cell genomics-based analysis reveals a vital ecological role of Thiocapsa sp. LSW in the meromictic Lake Shunet, Siberia. Microb Genom 7:712. 10.1099/MGEN.0.000712 10.1099/mgen.0.000712 PMC8767323 34860152

[CR62] Zander PD, Wirth SB, Gilli A et al (2023) Hyperspectral imaging sediment core scanning tracks high-resolution Holocene variations in (an)oxygenic phototrophic communities at Lake Cadagno, Swiss Alps. Biogeosciences 20:2221–2235. 10.5194/BG-20-2221-2023

