# Supplementary material for: Physically stable yet biologically sensitive: multiyear ecological dynamics of anoxygenic phototrophs in stably redox-stratified Lake Cadagno
Source: Aquat Sci. 2025 Apr 16;87(2):58. doi: 10.1007/s00027-025-01183-1 (PMC12003485; doi:10.1007/s00027-025-01183-1)
Supplement: Supplementary file 1 — Supplementary file1 (DOCX 1028 KB) [file 27_2025_1183_MOESM1_ESM.docx]

**Physically stable yet biologically sensitive: three years ecological dynamics of anoxygenic phototrophs in stably redox-stratified Lake Cadagno**

N. Storelli^1,2*^, O. Sepúlveda Steiner^3,4^, F. Di Nezio^1,2^, S. Roman^1,5^, A. Buetti-Dinh^1^, and D. Bouffard^3,6^

1: University of Applied Sciences and Arts of Southern Switzerland (SUPSI), Department of Environment, Constructions and Design, Institute of Microbiology, Via Flora Ruchat-Roncati 15, 6850 Mendrisio, Switzerland.

2: University of Geneva, Department of Plant Sciences, Boulevard d’Yvoy 4, 1205 Geneva, Switzerland.

3: Eawag, Swiss Federal Institute of Aquatic Science and Technology, Surface Waters – Research and Management, Seestrasse 79, 6047 Kastanienbaum, Switzerland.

4: Civil & Environmental Engineering, University of California – Davis, 3155 Ghausi Hall, Davis, CA 95616, USA.

5: Alpine Biology Center Foundation, Via Mirasole 22A, 6500 Bellinzona, Switzerland.

6: Institute of Earth Surface Dynamics, University of Lausanne, Quartier UNIL-Mouline, 1015 Lausanne, Switzerland.

*Corresponding author: Nicola Storelli ([nicola.storelli@supsi.ch](mailto:nicola.storelli@supsi.ch))


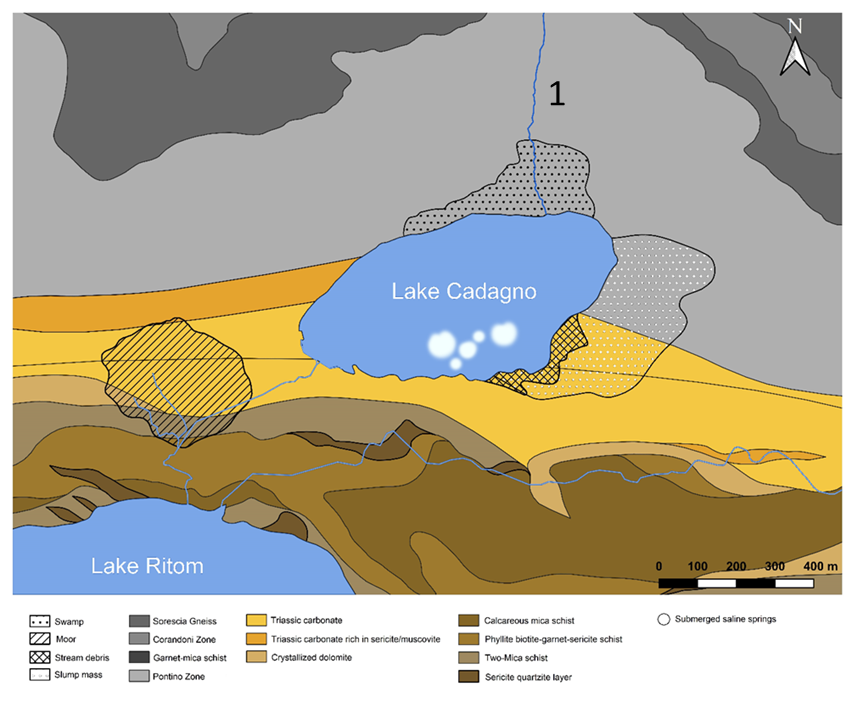


Figure S1. Overview of the geological composition of the Piora valley. The Piora-syncline is colored in yellow. The blue lines correspond to the streams, such as the one flowing from Stabbio Lake (1), which feeds crystal-clear water into the mixolimnion of Lake Cadagno. The “white spots” visible in Lake Cadagno are the entry points of sublacustrine springs that feed water enriched in salts, from dolomite, into the lake. Map created using QGIS® software (version 3.32.1). Data sourced from the Swiss Federal Office of Topography (<https://www.swisstopo.admin.ch/>).

**Monitoring profiles before 2016.**

Until 2015, physicochemical parameters of the water column were determined using a YSI 6000 profiler (Yellow Springs, Inc., Yellow Springs, OH) and included temperature (°C), conductivity (µS cm^-1^), pH, dissolved oxygen (mg l^-1^), redox potential (ORP; mV) and turbidity (FTU, formazine turbidity unit), in combination with an 800 ml Niskin bottle attached to a second winch with samples every meter in the mixolimnion and monimolimnion and 0.2 m in the chemocline, as shown in Figure S2 and past studies (Tonolla et al. 2003; Storelli et al. 2013).


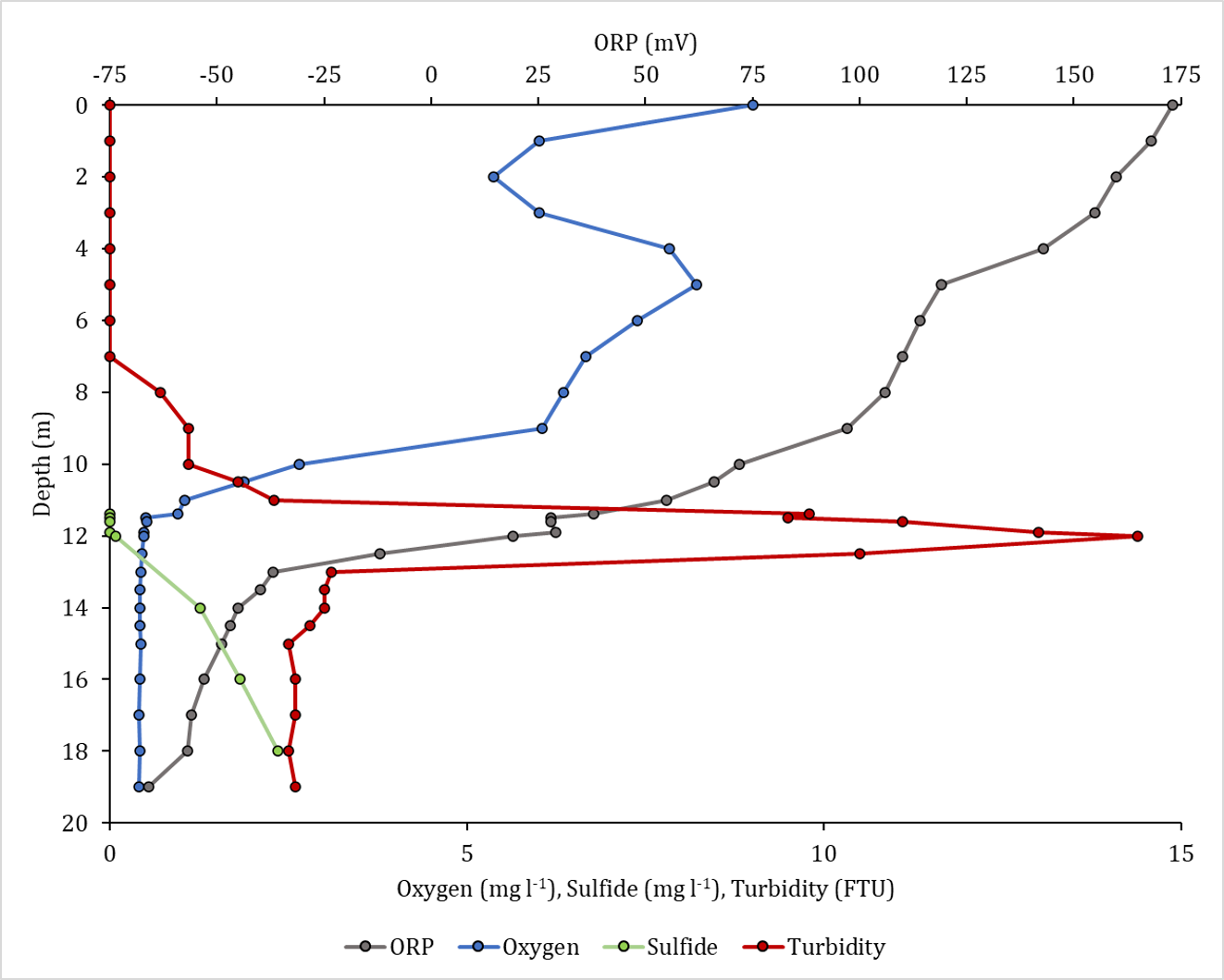


**Figure S2**. **Vertical profile of the Lake Cadagno water column**. Physicochemical profile of the water column of meromictic Lake Cadagno on 09 July 2019 measured with a YSI 6000 profiler (Yellow Springs, Inc., USA). In black is the profile of oxide reductive potential (ORP; mV), in blue that of dissolved oxygen (O_2_; mg l^-1^), in green that of sulfide (H_2_S; mg l^-1^), and in red that of dissolved particles (turbidity; Formazine Turbidity Unit FTU).

**Intense rainstorm events can alter the ecological dynamics of the BL.**

To assess the ecological system sensitivity to changes in weather, we analyzed samples specifically following 2020 rainfall events of varying intensity (Figure S3 and Table 2). To underscore the effect of the most intense rainstorm event in September 2020, data from a “typical September” of 2019 was overlaid as a comparison. These events were detected by evaluating the gross precipitation observed seven days before the BL sampling (Figure S3, gray bars).


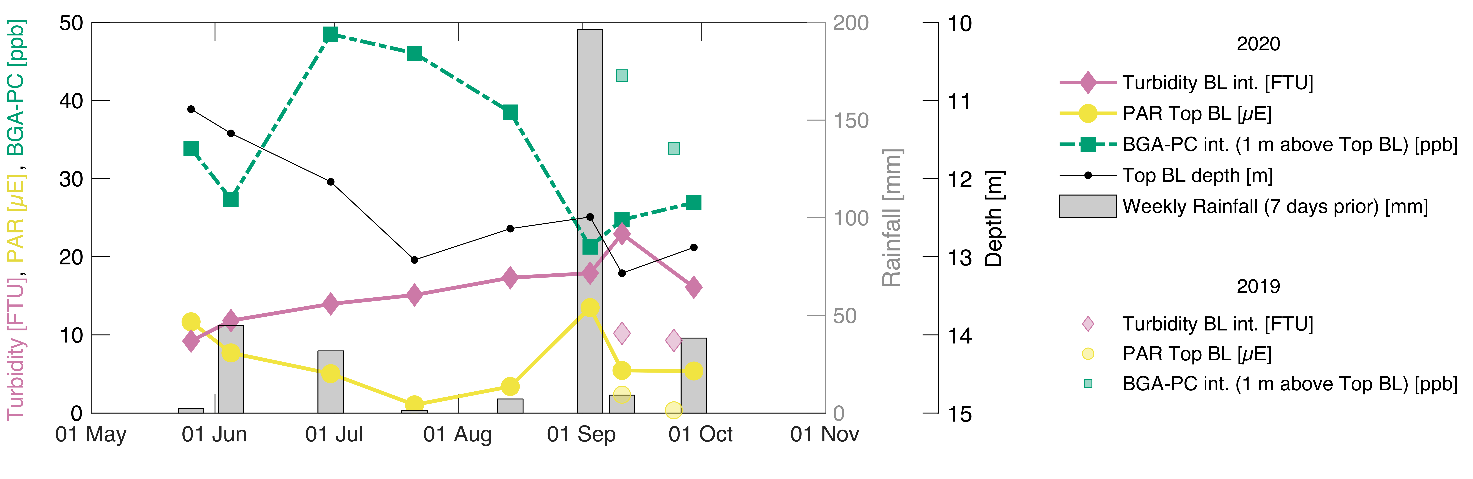


**Figure S3. Influence of rainstorm events on the chemocline microbial community**. The gray bars correspond to the amount of rainfall (mm total) measured in the seven days preceding the sampling day. Color lines with symbols indicate light values at the top of the BL (µE, yellow), turbidity values integrated through the entire BL (FTU, pink), and integrated concentration of blue-green algae up to 1.0 m above the BL (ppb, green) measured at the date indicated by the symbol. The position of the BL in the water column was defined by the depth where the turbidity exceeds 10 FTU (Top BL, black-dotted line). We included the values measured in September 2019 as control values to highlight the effect caused by an extreme rainstorm event (single blurred dots).

**Flow cytometry BL’s community counting.**

A BD Accuri C6 cytometer (Becton Dickinson, San Jose, CA) equipped with two lasers (488 and 680 nm), two scatter detectors and four fluorescence detectors (laser 488 nm: FL1 = 533/30, FL2 = 585/40, FL3 = 670; laser 640 nm: FL4 = 675/25) was used for samples analysis. A threshold of 2000 on FSC-H was applied to exclude most of the unwanted abiotic particles. Furthermore, a FL3-A >1100 threshold was applied to FL3 (red fluorescence) to discriminate cells emitting autofluorescence due to chlorophyll and bacteriochlorophyll. Phototrophic sulfur bacteria were enumerated by flowcytometry (FCM), measuring (bacterio)chlorophyll-like autofluorescence particle events as described in Danza et al. (2017 and 2018).


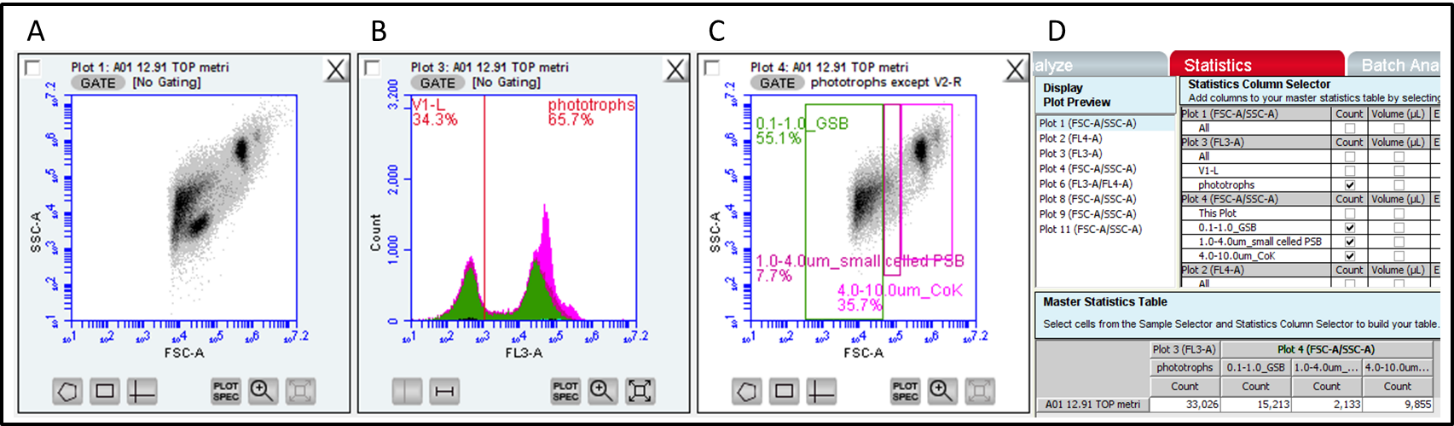


**Figure S4. The enumeration of the different phototrophic sulfur bacteria according to their size**. This image shows the different plots and gates used to differentiate the three cell types (phenotypes) present in the BL, here specifically is a sample taken at the beginning of the BL (TOP > 10 FTU) on 17 September 2020. All the “events” observed by the cytometer are shown in plots (A) differentiated by cell size (FSC) and cell complexity (SSC), and (B) with the number of events (count) as a function of florescence (FL3). The use of florescence allows differentiation between pigment-provided organisms (e.g., phototrophs) and the rest. Plot C considers only cells with a pigment-driven self-florescence (phototrophs 65.7 %) and shown according to cell size (FSC) and complexity (SSC). Based on cytometer specifications and cell size, we defined 3 specific gates for cells between 0.1 and 1.0 um (GSB), 1.0 and 4.0 um (small PSB), and between 4.0 and 10.0 um (*C. okenii*), based on microscopic observations from previous studies (Tonolla et al. 2005; Musat et al. 2008; Danza et al. 2017; Di Nezio et al. 2021). Part D of the figure then shows the number of events for each gate used to create the graphs in Figure 4, which are available as raw data in Zenodo (10.5281/zenodo.10663093).
